# Bioelectrical Analysis of Various Cancer Cell Types Immobilized in 3D Matrix and Cultured in 3D-Printed Well

**DOI:** 10.3390/bios9040136

**Published:** 2019-11-14

**Authors:** Georgia Paivana, Sophie Mavrikou, Grigoris Kaltsas, Spyridon Kintzios

**Affiliations:** 1 Laboratory of Cell Technology, Department of Biotechnology, Agricultural University of Athens, 118 55 Athens, Greece; georpaiv@gmail.com (G.P.); skin@aua.gr (S.K.); 2 microSENSES Laboratory, Department of Electrical and Electronic Engineering, University of West Attica, 122 44 Athens, Greece; G.Kaltsas@uniwa.gr

**Keywords:** cell immobilization, 3D-printed well, bioelectric profiling, impedance analysis, real-time measurements

## Abstract

Cancer cell lines are important tools for anticancer drug research and assessment. Impedance measurements can provide valuable information about cell viability in real time. This work presents the proof-of-concept development of a bioelectrical, impedance-based analysis technique applied to four adherent mammalian cancer cells lines immobilized in a three-dimensional (3D) calcium alginate hydrogel matrix, thus mimicking in vivo tissue conditions. Cells were treated with cytostatic agent5-fluoruracil (5-FU). The cell lines used in this study were SK-N-SH, HEK293, HeLa, and MCF-7. For each cell culture, three cell population densities were chosen (50,000, 100,000, and 200,000 cells/100 μL). The aim of this study was the extraction of mean impedance values at various frequencies for the assessment of the different behavior of various cancer cells when 5-FU was applied. For comparison purposes, impedance measurements were implemented on untreated immobilized cell lines. The results demonstrated not only the dependence of each cell line impedance value on the frequency, but also the relation of the impedance level to the cell population density for every individual cell line. By establishing a cell line-specific bioelectrical behavior, it is possible to obtain a unique fingerprint for each cancer cell line reaction to a selected anticancer agent.

## 1. Introduction

Cancer is the main cause of death in many countries, as it appears in different types, most commonly affecting women (e.g., cervical, breast, and lung adenocarcinoma cancers) [1,2]. In medical treatment, doctors take steps to anticipate the development of the disease (primary prophylaxis) or to minimize its further development (secondary prophylaxis) [3]. Considering secondary prophylaxis measures, sophisticated processes are required to detect possible cellular disorders at the very earliest stages of the disease’s incubation period, taking into account the dependence of the timeliness with which the disease is detected [4]. Due to the fact that many cancer diagnostic methods combined with radiological, surgical biopsy, and pathological assessments of tissue samples based on immunohistochemical and morphological characteristics [5] are time-consuming, invasive, and complicated, and require rigorous laboratory conditions, new cancer detection methods are being developed which are minimally invasive, more reliable, cheaper, and easier to use [6].

Chemotherapy, newer immunotherapy, and targeted therapy constitute the various treatment strategies that have been proposed and modified in order to increase effectiveness and precision [7,8]. Although many approaches for cancer treatments have been developed successfully, sooner or later, resistance among the subgroups of cancer cells will emerge as a hurdle to the efficacy of most current therapeutic approaches [9]. One of the most commonly used drugs for cancer treatment is 5-fluorouracil (5-FU). This compound is used in the treatment of many types of cancer, including breast cancer, colon cancer, skin cancer, etc., as it intercalates in nucleoside metabolism, leading to cytotoxicity and cell death [10].

Electrical impedance spectroscopy (EIS) is a technique that measures the electrical impedance of living cells in order to identify various cell types. This technique can be used for the successful separation of pathological cells from normal ones, taking into account the electrophysiological properties of cells based on the frequency range [11,12]. Cell electrical impedance can identify the physical, mechanical, and biochemical functions of living biological cells. EIS focuses on the analysis and discrimination of cancer cells, utilizing the fact that impedance measurements represent an effective approach for cell characterization based on the electrical responses over a particular frequency domain. As the impedance value of several tissue parameters (e.g., morphology, growth, and differentiation) vary with the frequency of the applied signal, an impedance analysis conducted over a wide frequency range provides more information about the tissue interiors, which helps us to better understand the biological tissues physiology, anatomy, and pathology [13]. For example, researchers have used the impedance technique to measure three dimensional cell cultures for four breast cell lines [14,15,16].

Recently, bioimpedance has been able to provide in-depth biological measurement analyses from the cell-level to DNA [17,18]. The evaluation of parameters such as cell adhesion, differentiation, spreading, morphology, growth, motility, and death for any adherent cell type is possible by monitoring the impedance changes at the contact point between the cells and electrodes [19]. In addition, bioimpedance research can nominate the pathological status of a single cell, and also be used to determine the occurrence of bacterial infections, toxicity, and changes of environmental parameters, and in the direct or indirect detection of compounds and other factors [20]. Critical changes in cellular behavior, such as the integrity of the extracellular membrane, morphology, as well as alterations in intracellular structure, significantly influence the corresponding impedance level which can be detected quickly and cost-effectively using electrodes [13,19]. Thus, impedance measurements can also be used in studying cell viability, which provides an alternative to slow and invasive traditional cytotoxicity assays [21].

Biomaterial research for drug development, cell culture, and tissue regeneration applications aims to mimic the natural extracellular matrix (ECM) in order to bridge the gap between in vivo and in vitro environments [22]. In the body, nearly all tissue cells are supported by an ECM that comprises a complex, three-dimensional (3D), fibrous mesh network of collagen and elastic fibers integrated into a high hydrated, gel-like material containing proteoglycans, glycosaminoglycans, and glycoproteins. This complex system is responsible for the triggering of various biochemical and physical signals [23]. In practice, most cell culture studies are carried out using cells cultured as two-dimensional (2D) monolayers on hard plastic surfaces due to the convenience, ease, and high cell proliferation that these culture techniques provide. On the other hand, cell adaption to an artificial monolayer culture on an inflexible surface would lead to metabolic and functional alterations, resulting in behavior different from the in vivo environment [23]. Thus, research is focused on developing more controllable 3D cell culture matrices resembling, as much as possible, the in vivo conditions that are able to support cell growth, differentiation, and organization. Bearing in mind all the above, we can define 3D cell culture as the integration of cells into a hydrogel matrix in order to receive signals from the scaffold and surrounding cells [23,24]. This procedure initially necessitates a cellular suspension in a hydrogel precursor solution, and then entrapment through a gel initiation reaction that leads to the formation of covalently- or noncovalently-linked molecules [25,26].

A great number of synthetic and natural polymers can be used for cell entrapment gelled into hydrophilic matrices under mild conditions with minimal loss of viability [27]. The properties of the gel, i.e., either hydrophobic either hydrophilic, and its porosity, can be regulated. The entrapment of cells constitutes one of the most widely used methods for living cell immobilization within spherical beads of calcium alginate. This method is considered successful due to the fact that immobilization is a simple, quick and cost-effective technique, and is usually performed under very mild conditions [28].

Three-dimensional (3D) printing is a cheap additive layer manufacturing technology that is able to create sophisticated and complex-shaped bodies in a short time, especially for rapid prototyping engineering applications [29,30,31]. In many cases, 3D printing technology uses polymers, depending on the specific characteristics that are required for the microfabricated devices (e.g., polycarbonate (PC), PLA, nylon, polymethyl methacrylate (PMMA), polystyrene, and polyethylene terephthalate glycol (PETG)) [32,33,34,35]. Depending on the area of interest, 3D printing technology is increasing rapidly and is extensively used in many fields, such as bio-printing, medical devices, the automotive industry, soft sensors and actuators, space, art and jewelry, education, and tissue printing [36,37]. PETG constitutes a copolymer known for its biocompatibility, chemical resistance, recyclability, and transparency [38]. It can be used in different applications in the food and medical industry, with an acceptable flammability rating [39]. Unfortunately, it presents low resistance to ultraviolet (UV) light and performs weakly against frictional contact and scratching.

The aim of this study is to develop a proof-of-concept bioelectrical profiling assay to study the reaction of various cancer cell lines exposed to a common anticancer drug as a function of the cell population density. For comparison purposes, bioelectrical impedance-based measurements were taken on both untreated immobilized cells and on cells treated with 5-FU. Thus, two gold-plated (Au) electrodes were embedded in a 3D-printed PETG well for impedance measurements on four cancer cell lines (SK-N-SH, HEK293, HeLa and MCF-7) immobilized in calcium alginate matrix. Cell cultures were realized in three population densities tested with various frequencies. In this way, a more detailed application of the bioelectrical analysis on an in vitro system for monitoring different responses between various cancer cells (control and treated with 5-FU) was possible.

## 2. Materials and Methods

### 2.1. Theory

Bioimpedance, as a passive electrical property, is described as the capability of biological tissue to impede electric current. Bioimpedance measurements detect the response to electrical activity (potential or current). Bioimpedance is a complex quantity, mainly determined by the resistance (R) of the total amount of body water and by the capacitance of the cell membrane [40]. Electrical Impedance (Z) is defined by the ratio of the voltage (V) to the current (I), and is quite similar to resistance. The basic difference is that impedance extends to the frequency domain, and thus, is used in AC circuits, while resistance mainly refers to DC applications. The equation for the calculation of electrical impedance is:

Z = V/I(1)

The determination of electrical impedance requires not only the application of an alternating current across a biological tissue, but also the measurement of the consequential differential voltage of the tissue sample, described in the following equations:

I(ω) = Ι_0_·cos(ωt + θ) (2)

V(ω) = V_0_·sin(ωt + ψ) (3)

where I_0_ and V_0_ represent the amplitude θ and ψ the phase of the current and voltage signal, respectively. Taking into account that both I_0_ and V_0_ are calculated at the same angular frequency, i.e., ω = 2πf, electrical impedance can be described as Z(ω) = (V(ω))/(I(ω)) [41]. The expression of Z (Equation (1)) as a complex function can be used either as the modulus of the absolute value and the phase shift, or as the real part, R, representing resistance, and the imaginary part, X, representing capacitance and inductance, respectively [42]. In the case of direct current (DC) application, the imaginary part would be zero. The inverse of the impedance is called admittance (Y) and describes the current flow. Impedance and admittance constitute AC parameters, and both are frequency dependent. The EIS measurement procedure involves the characterization of the complex impedance over a wide range of frequencies, as shown in Equation (3):

Z(ω) = |Z|(cos φ + j sin φ) = R + j X (4)

### 2.2. Cell Culture

SK-N-SH neuroblastoma cells (ATCC^®^ HTB-11™) were cultured under standard conditions (37 °C, 5% CO_2_) in 90% Minimum Essential Medium (MEM) (Eagle) with Earle’s balanced salt solution (BSS) (Biowest, Nuaillé, France) and fetal bovine serum (FBS) (Thermo Fisher Scientific, Waltham, MA, USA) to a final concentration of 10%, 2 mM l-glutamine, 1.5 g/L sodium bicarbonate, 0.1 mM non-essential amino acids, 1 U μg^−1^ antibiotics (penicillin/streptomycin), and 1.0 mM sodium pyruvate (Biowest, Nuaillé, France). HEK293 (ATCC^®^ CRL-1573™), HeLa (ATCC^®^ CRM-CCL-2™) and MCF-7 (ATCC^®^ HTB-22™) cell lines were grown in Dulbecco’s Modified Eagle Medium (Biochrom Gmbh, Berlin, Germany), supplemented with 10% Fetal Bovine Serum (Thermo Fisher Scientific, Waltham, MA, USA), 2 mM l-glutamine, 0.5 mM sodium pyruvate, and 1% Penicillin-Streptomycin (Biowest, Nuaillé, France) in T-75 flasks (Sarstedt AG & Co. KG, Nümbrecht, Germany). Subcultivation was done in a 1:10 ratio. Cells were detached from culture flasks by treatment with trypsin-EDTA for 3–10 min. After detachment, they were resuspended in the culture medium to inactivate any remaining trypsin activity. After centrifugation for 5 min (1000 rpm), they were resuspended in the medium at concentrations of 10^6^, 2 × 10^6^, and 4 × 10^6^ cells/mL.

### 2.3. Cells Preparation/Immobilization

Cell immobilization was performed in calcium alginate as an immobilization matrix. Briefly, sodium alginate in 1.5% concentration, sterilized by autoclave (121 °C, 20 min), was mixed with 5 × 10^4^, 10^5^, and 2 × 10^5^ cells to a 0.75% final concentration and poured together in the well. Then, a 1% CaCl_2_ gelling solution was added for 10 s for cross-linking, and washed with phosphate buffered saline (PBS). After washing, the calcium alginate scaffolds with the cells were incubated in the culture medium. The next day, the culture medium was removed and replaced with 1% FBS medium and 1% FBS with different concentrations 5-FU (Sigma-Aldrich Chemie GmbH, Taufkirchen, Germany).

### 2.4. Cell Viability Assay

Cell viability was evaluated by a 3-(4, 5-dimethylthiazol-2-yl)-2,5-diphenyltetrazolium bromide (MTT) colorimetric assay [43] with 5-FU as the positive control. The concentration of 5-FU for each cell line, indicated in Table 1, was selected based on previously published data [44,45,46,47,48]. These concentrations gave at least 30% inhibition in cell proliferation after 24 h incubation. The next day, the cells were treated with 0.5 mg/mL MTT (Duchefa Biochemie, Haarlem, The Netherlands) and incubated with dye for 3 h. After incubation, the medium was removed and the cell containing alginate scaffolds were solubilized with 0.1 M ethylenediaminetetraacetic acid per well. Cell morphology observations were performed with an inverted microscope (ZEISS Axio Vert.A1, Carl Zeiss Microscopy, LLC, White Plains, NY, USA), and pictures were processed using the ZEN lite software. The optical absorbance was measured with a PowerWave240 plate reader (BioTek, Winooski, VT, USA) at 560 nm. The experiment was repeated independently three times for each treatment, and the mean results were expressed as the average OD for each treatment. All values were presented as means ± SD, and significance testing in the comparisons was based on Student’s T-tests for pairs, as they did not follow a normal distribution. The Student’s T-test gives the probability that the difference between the two means is caused by chance. *p* values < 0.05 were considered to be statistically significant.

### 2.5. Experimental Setup

**Figure 1:**
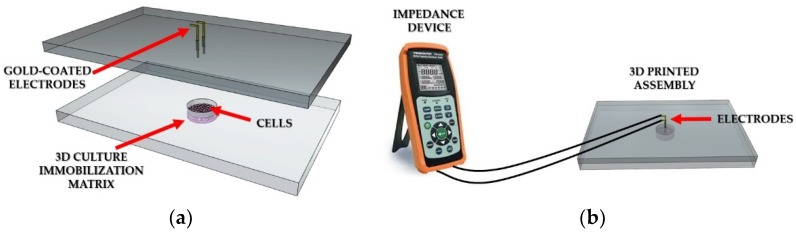
Experimental setup. (**a**) Representation of the cell chamber filled with 3D cell immobilization matrix; (**b**) Connection of the LCR meter to the 3D printed well.

In order to perform the bioelectrical measurements, a specifically designed electrode-based system was fabricated. Figure 1a illustrates the experimental setup of the system used for the measurement procedure. More specifically, two gold-coated (Au) electrodes were placed vertically into a custom-made, transparent, 3D-printed PETG well that was designed to be used as a cell cultivation vessel. The application of 3D printing technology to the assembly of culture systems can provide an appropriate environment for cell growth which is able to mimic physiological and realistic cell phenotypes [49]. The well model was designed using the 123D Design software (Autodesk, San Rafael, CA, USA). A Cel Robox 3D printer device was utilized for the printing procedure, applying the fused deposition modeling technique (FDM). By this method, the filament that passes through the heated print head is laid on a construction platform in a layer-by-layer fashion until the object’s form is complete [50]. The nozzle diameter of the print head was 0.4 mm and the printing temperature for PETG was 190 °C [38]. The PETG filament was obtained by the Formfutura BV (Nijmegen, The Netherlands); the filament diameter was 1.75 ± 0.05 mm. After the printing process, the wells were sterilized with 70% (v/v) ethanol for 10 min, and then dried for 2 h under a sterile hood. The electrodes were connected to the handheld LCR meter U1733C (Figure 1b) from Keysight Technologies (Santa Rosa, CA, USA); the instrument is able to measure at three frequencies (1 KHz, 10 KHz, and 100 KHz) for the direct extraction of impedance magnitude of the sample tested. For impedance measurements, a voltage of 0.74 V_rms_ ± 50 mV_rms_ was applied via the two terminals to the gold-coated electrodes. The best sampling rate of the instrument was 1 Hz (one measurement per sec); each measurement lasted one minute; thus, the total values obtained for each run were 60, with a measurement frequency of 1 Hz. All data were normalized values, presented as the mean of the absolute (ABS) value of the control (plain cell culture medium) minus the absolute value of cells, for both cases (treated or untreated with 5-FU ± SD), as shown in Equation (5). Significance testing in comparisons was based on Student’s T-tests for pairs, and *p* values < 0.05 were considered to be statistically significant.

Normalized value = mean (|control-cell value|) (5)

## 3. Results

In this study, we evaluated the applicability of impedance measurements for the bioelectric profiling of different cancer cell types treated with substance-selected anticancer agents. More specifically, four cancer cell lines were immobilized in calcium alginate and cultured in different cell population densities (50,000, 100,000, and 200,000/100 μL). Then, 5-fluorouracil (5-FU) was applied, as it constitutes one of the most common cancer therapeutic drugs. In each case, three frequencies were tested: 1 KHz, 10 KHz, and 100 KHz.

### 3.1. Cell Proliferation

**Figure 2:**
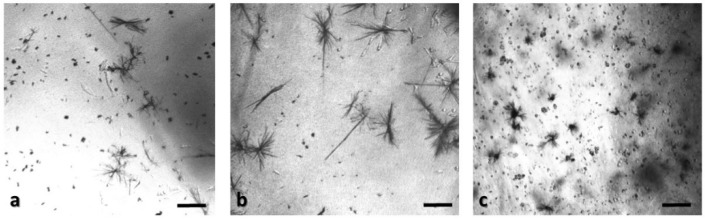
Panoramic view of SK-N-SH immobilized cells in 3D matrix after treatment with MTT for 24 h, showing the viability in three different population densities: (**a**) 50,000 cells; (**b**) 100,000 cells; and (**c**) 200,000 cells. Scale bars = 50 μm.

**Figure 3:**
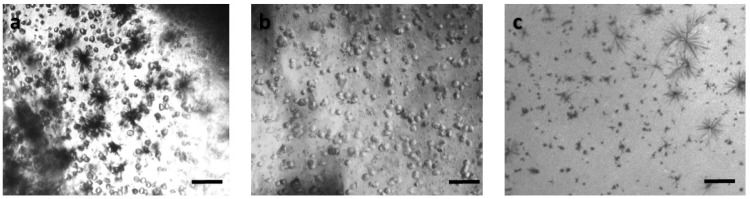
Panoramic view of HEK293 immobilized cells in 3D matrix after treatment with MTT for 24 h showing the viability in three different population densities: (**a**) 50,000 cells; (**b**) 100,000 cells; and (**c**) 200,000 cells. Scale bars = 50 μm.

**Figure 4:**
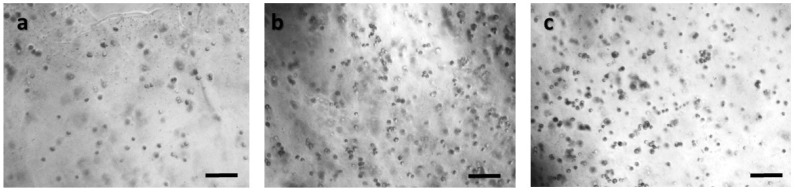
Panoramic view of HeLa immobilized cells in 3D matrix after treatment with MTT for 24 h showing the viability in three different population densities: (**a**) 50,000 cells; (**b**) 100,000 cells; and (**c**) 200,000 cells/100 μL. Scale bars = 50 μm.

**Figure 5:**
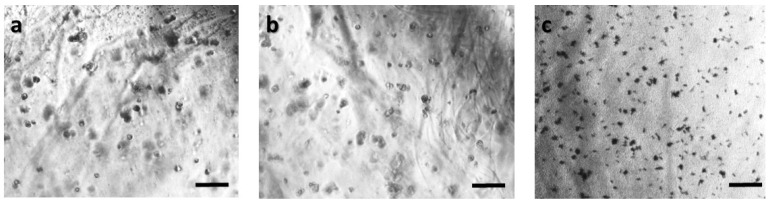
Panoramic view of MCF-7 immobilized cells in 3D matrix after treatment with MTT for 24 h showing the viability in three different population densities: (**a**) 50,000 cells; (**b**) 100,000 cells; and (**c**) 200,000 cells/100 μL. Scale bars = 50 μm.

In order to ensure that calcium alginate was a proper immobilization matrix for the cancer cell culture, we assessed cellular viability with the MTT uptake assay. Cells were cultured in the matrix for 24 h (with and without treatment with 5-FU), and the proliferation was determined microscopically and photometrically after MTT application. Figure 2, Figure 3, Figure 4 and Figure 5 depict the microscopic observations for three different populations of the four cell lines immobilized in calcium alginate after incubation with MTT.

**Figure 6:**
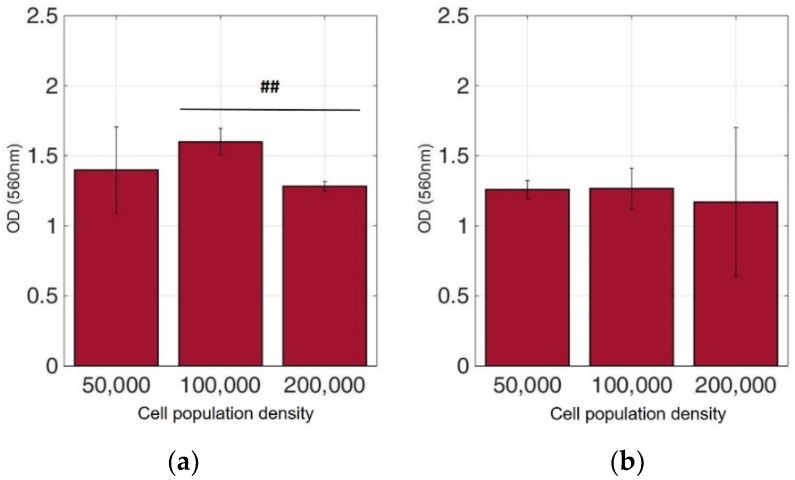
Cellular viability of SK-N-SH cells immobilized in 3D matrix after treatment with MTT for 24 h showing the viability in three different population densities (50,000, 100,000, and 200,000 cells/100 μL) ± STD: (**a**) untreated cells (control); (**b**) cells treated with 5-FU. ## < 0.01 significantly different from 100,000 cells/100 μL.

**Figure 7:**
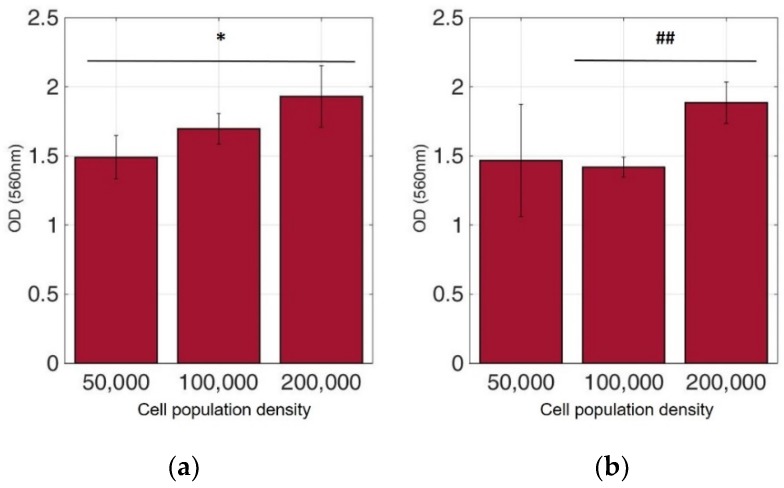
Cellular viability of HEK293 cells immobilized in 3D matrix after treatment with MTT for 24 h showing the viability in three different population densities (50,000, 100,000, and 200,000 cells/100 μL) ± STD: (**a**) untreated cells (control); (**b**) cells treated with 5-FU. * < 0.05 significantly different from 50,000 cells, ## < 0.01 significantly different from 100,000 cells/100 μL.

**Figure 8:**
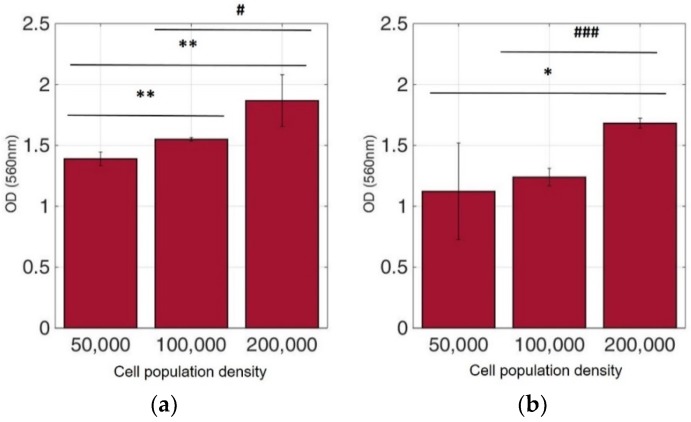
Cellular viability of HeLa cells immobilized in 3D matrix after treatment with MTT for 24 h showing the viability in three different population densities (50,000, 100,000, and 200,000 cells/100 μL) ± STD: (**a**) untreated cells (control); (**b**) cells treated with 5-FU. * < 0.05, ** < 0.01 significantly different from 50,000 cells, # < 0.05, ### < 0.001 significantly different from 100,000 cells/100 μL.

**Figure 9:**
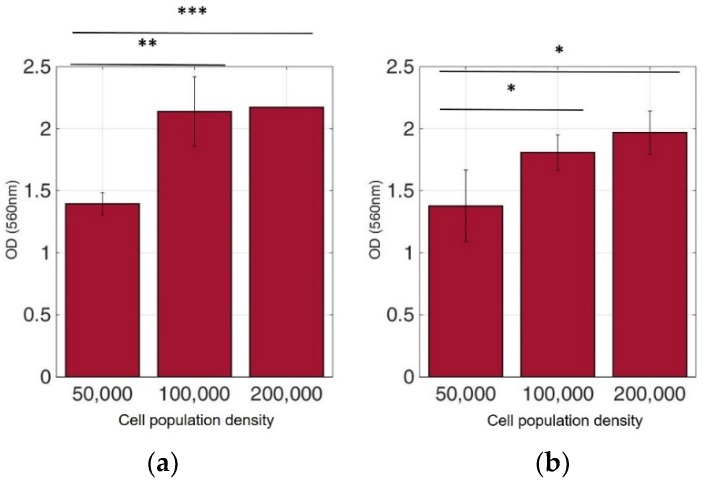
Cellular viability of MCF-7 cells immobilized in 3D matrix after treatment with MTT for 24 h showing the viability in three different population densities (50,000, 100,000, and 200,000 cells/100 μL) ± STD: (**a**) untreated cells (control); (**b**) cells treated with 5-FU. * < 0.05, ** < 0.01, *** < 0.001 significantly different from 50,000 cells.

Viable cells were dyed purple using the yellow formazan (MTT) by intracellular NAD(P)H-oxidoreductases [43]. We can see that cellular proliferation is affected neither by the immobilization matrix, nor by the increase in the cell population density. Contrary to this observation, the results from the photometric MTT determination presented in Figure 6, Figure 7, Figure 8 and Figure 9 showed an increase in the absorbance as cell number population densities increase, whereas the addition of 5-FU led to a significant reduction in cell viability (see Table 2) in almost all cell lines. Cell population alterations in the neuroblastoma SK-N-SH cell line (see Figure 6) appear to have a limited impact in MTT absorbance for both cell cases, i.e., treated with 5-FU and untreated. On the other hand, in the case of the remaining cell lines (Figure 7, Figure 8 and Figure 9), we observed an increase in absorbance proportional to the cell number.

For further analysis, all cell line combinations were compared using a Student’s T-test in each population density, with or without the addition of 5-FU. As shown in Table 3, in the 50,000 cell/100 μL population density, treatment with 5-FU did not significantly affect MTT uptake. However, it seems that the other two population densities contributed to differential viability results, i.e., with or without treatment with 5-FU.

### 3.2. Comparative Bioelectrical Profiling Results among Different Immobilized Cell Lines

**Figure 10:**
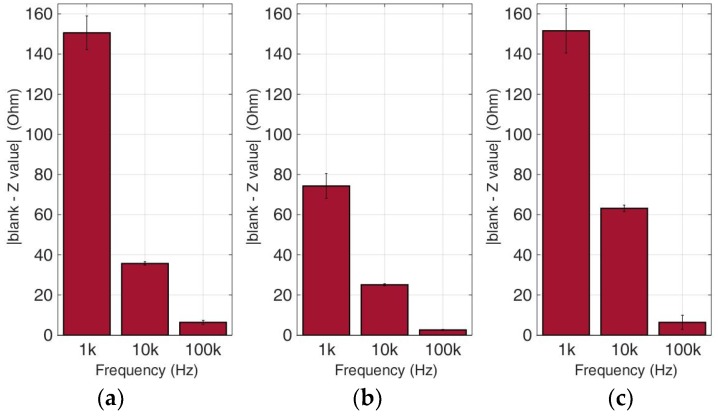
Normalized values of the mean impedance magnitude for untreated (control) immobilized SK-N-SH cancer cell lines tested at three frequencies (1 KHz, 10 KHz, 100 KHz) for three different population densities ± STD: (**a**) 50,000 cells; (**b**) 100,000 cells and (**c**) 200,000 cells/100 μL.

**Figure 11:**
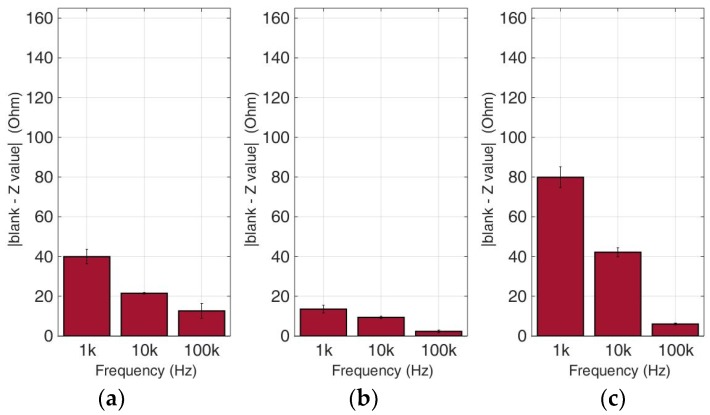
Normalized values of the mean impedance magnitude for untreated (control) immobilized HEK293 cancer cell lines tested at three frequencies (1 KHz, 10 KHz, 100 KHz) for three different population densities ± STD: (**a**) 50,000 cells; (**b**) 100,000 cells and (**c**) 200,000 cells/100 μL.

**Figure 12:**
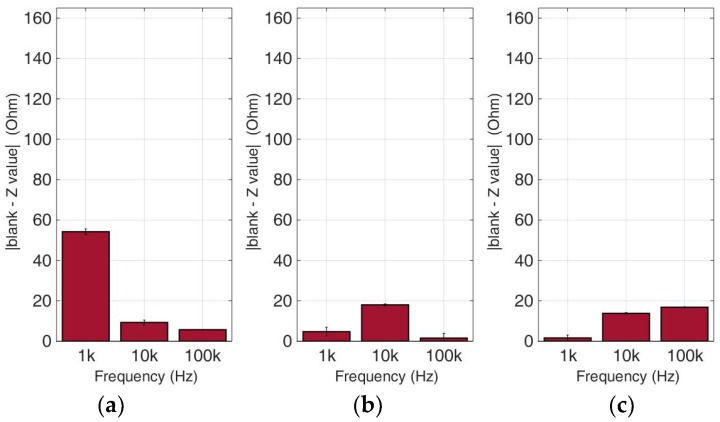
Normalized values of the mean impedance magnitude for untreated (control) immobilized HeLa cancer cell lines tested at three frequencies (1 KHz, 10 KHz, 100 KHz) for three different population densities ± STD: (**a**) 50,000 cells; (**b**) 100,000 cells and (**c**) 200,000 cells/100 μL.

**Figure 13:**
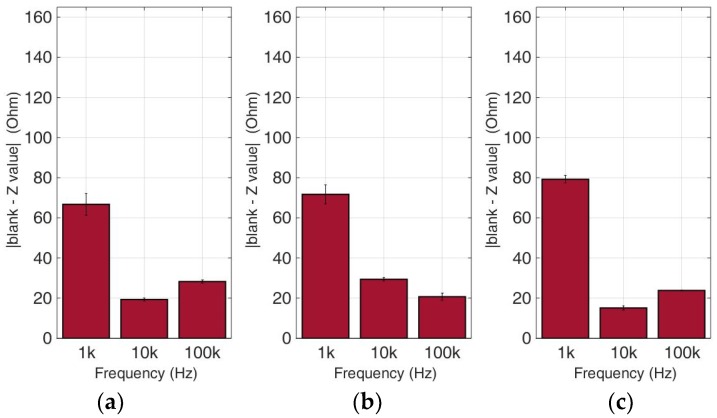
Normalized values of the mean impedance magnitude for untreated (control) immobilized MCF-7 cancer cell lines tested at three frequencies (1 KHz, 10 KHz, 100 KHz) for three different population densities: (**a**) 50,000 cells; (**b**) 100,000 cells and (**c**) 200,000 cells/100 μL.

This experimental approach refers to the analysis of bioelectrical impedance-based measurements on various cancer cell types in different population densities. Calcium alginate was once again chosen as the 3D immobilization matrix used for each cancer cell culture. Figure 10, Figure 11, Figure 12 and Figure 13 depict the absolute values of the differences between the mean blank values and the mean impedance values for three population densities tested for each cell line chosen in three different frequencies (1 KHz, 10 KHz, and 100 KHz).

As indicated by many studies, and also in our case, a frequency-dependent impedance response is observed. Each cell line x population density combination corresponds to a different pattern in the impedance magnitude measured in each frequency. Thus, in neuroblastoma SK-N-SH cells (see Figure 10), we can observe that the normalized impedance value drops when we move from 50,000 to 100,000 cells, and then increases at 200,000 cells/100 μL at each frequency. A similar pattern is observed in the case of HEK293 cells, but as shown in Figure 11, at 200,000 cells/100 μL population density, the impedance value is higher than the respective impedance in 50,000 cells. However, HeLa and MCF-7 cell cultures (Figure 12 and Figure 13) appear to have a totally different behavior. More specifically, at a lower frequency (1 KHz), HeLa cells initially demonstrated a high impedance value (54, 23 Ohm), and as population density increased, a dramatic decrease was observed (1, 60 Ohm). In contrast, MCF-7 cells demonstrated the opposite trend, starting with 66, 72 Ohm at 50,000 cells and ending up at 79, 23 Ohm at 200,000 cells/100 μL. Furthermore, at the other two frequencies, we observed differential fluctuations for both cell lines. In other words, each cell line x population density combination was characterized by its own unique impedance behavior fingerprint.

### 3.3. Comparative Bioelectrical Profiling Results among Different Immobilized Cell Lines Treated with 5-FU

**Figure 14:**
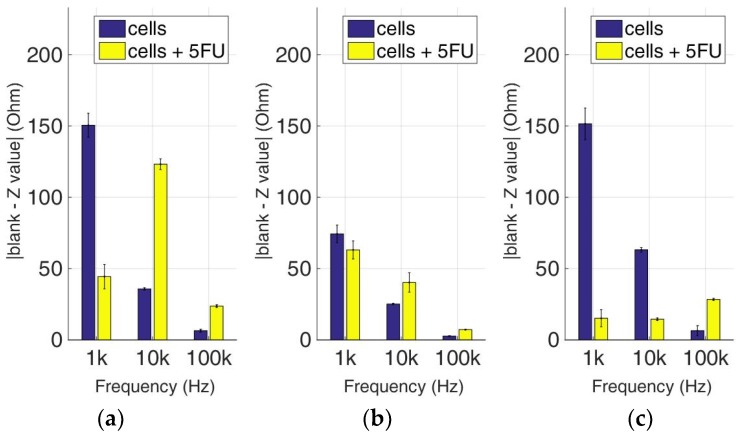
Normalized values of the mean impedance magnitude for control immobilized SK-N-SH cells (blue bars) and immobilized SK-N-SH cells treated with 5-FU (yellow bars), tested at three different cell population densities ± STD: (**a**) 50,000; (**b**) 100,000 and (**c**) 200,000/100 μL for three different frequencies (1 KHz, 10 KHz, and 100 KHz).

**Figure 15:**
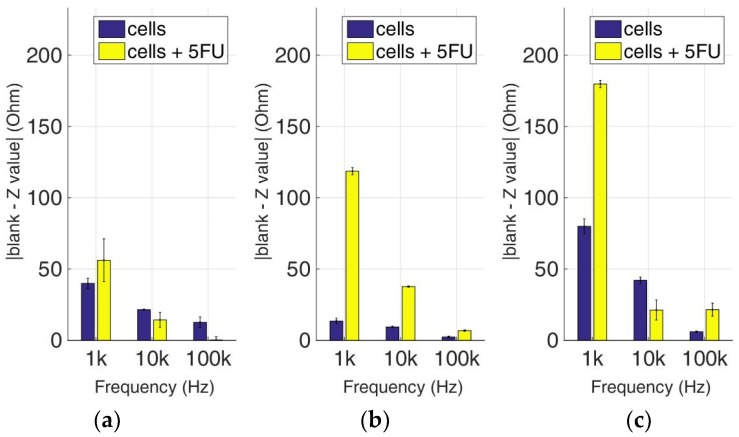
Normalized values of the mean impedance magnitude for control immobilized HEK293 cells (blue bars) and immobilized HEK293 cells treated with 5-FU (yellow bars), tested at three different cell population densities ± STD: (**a**) 50,000; (**b**) 100,000 and (**c**) 200,000/100 μL for three different frequencies (1 KHz, 10 KHz, and 100 KHz).

**Figure 16:**
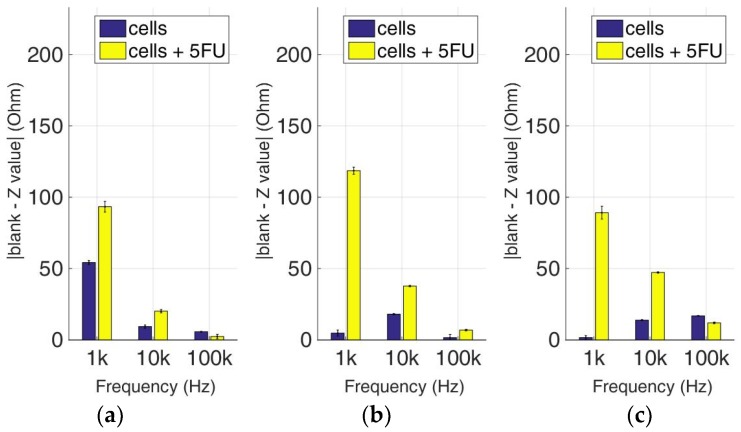
Normalized values of the mean impedance magnitude for control immobilized HeLa cells (blue bars) and immobilized HeLa cells treated with 5-FU (yellow bars), tested at three different cell population densities ± STD: (**a**) 50,000; (**b**) 100,000 and (**c**) 200,000/100 μL for three different frequencies (1 KHz, 10 KHz, and 100 KHz).

**Figure 17:**
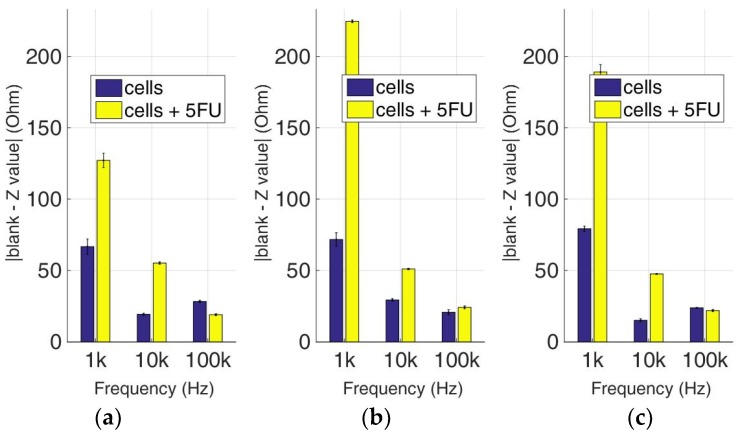
Normalized values of the mean impedance magnitude for control immobilized MCF-7 cells (blue bars) and immobilized MCF-7 cells treated with 5-FU (yellow bars), tested at three different cell population densities ± STD: (**a**) 50,000; (**b**) 100,000 and (**c**) 200,000/100 μL for three different frequencies (1 KHz, 10 KHz, and 100 KHz).

At this experimental stage, the evaluation of 5-FU applied to the previous immobilized cancer cell cultures was implemented to investigate the effects of this widely-used anticancer medicine through impedance measurements at specific frequencies. Figure 14, Figure 15, Figure 16 and Figure 17 depict the normalized impedance values after the subtraction of the mean blank values from the mean impedance values in three population densities tested for each cell line chosen in three different frequencies, with (yellow bars) or without (blue bars) 5-FU. The statistical significances after pair comparisons for all combinations of the cell populations are represented in Table 4, Table 5, Table 6 and Table 7.

When the anticancer agent 5-fluorouracil was applied for 24 h, we observed that in most cases (18 out of 27), 5-FU treatment gave higher impedance normalized values in comparison to cells with no treatment. The SK-N-SH cell line (see Figure 14) followed mostly the opposite pattern when 5-FU was applied compared to the rest of the cell lines, especially at a frequency of 1 KHz. In the case of 200,000 cells/100 μL population density, the cell impedance magnitude followed a downward trend, as opposed to cells under 5-FU exposure, that gave higher values in the frequency range of 1–100 KHz.

Figure 15 summarizes the results for the HEK293 cell line. Similar to the aforementioned observations, impedance significantly dropped with an increase in frequency in all population densities, and treatment with 5-FU led to higher normalized values (see Table 5). An exception can be observed at the frequencies of 10 KHz and 100 KHz in 50,000 cell population density, and also at the frequency of 10 KHz in 200,000 cells/100 μL population density, where the values obtained from control cells were higher than the respective values with 5-FU.

As depicted in Figure 16, HeLa cells treated with 5-FU follow a frequency-dependent, downward motif for every population density. On the other hand, untreated cells do not show the same pattern, since a downward trend is observed at 50,000 cells/100 μL, followed by an upward tendency at 200,000 cells/100 μL, and general non-linear behavior at 100,000 cells/100 μL. Once again, in almost all cases, the response of the cells treated with 5-FU is significantly higher in comparison with control cells (Table 6). The only exception is observed in 50,000 and 200,000 cells/100 μL population densities at 100 KHz. The latter frequency gave low impedance values for both the treated and untreated cell populations.

In the MCF-7 cell line (Figure 17), we observed particularly high impedance values when 5-FU was applied for every population density tested when compared with untreated cells, especially at a frequency of 1 KHz. These values significantly increased with population density at the same frequency (Table 7). The impedance values of cells not treated with 5-FU depicted low variations between different cell population densities. A general observation is that as frequency magnitude increases, the normalized impedance values decrease.

## 4. Discussion

Up to now, the development of cancer diagnostics was primarily controlled by direct tumor tissue biopsies for either pathologic and/or histologic analyses. Novel advanced molecular biology techniques such as next-generation DNA sequencing and genomics bioinformatics analysis represent examples of the transition from traditional microscopy of tissue samples to molecular genomics for cancer diagnosis. In combination with remarkable advances in drug development and efficiency, these exciting new trends have contributed to the transition to the era of personalized cancer diagnosis and therapy. Therefore, these advances in cancer management and treatment have incentivized the pursuit of avant-garde, non-invasive approaches for accurate detection and monitoring.

For this purpose, in this study, we investigated the differences between the electrical properties of different in vitro 3D cancer cell cultures such as cervical, breast, and kidney tumor models via impedance evaluation at various frequencies. In general, several variances are observed in cell activities such as morphology, proliferation, and gene and protein expression due to the additional dimensionality of 3D culture, compared to the 2D planar culture [51]. Hydrogels such as alginate (SA), collagen (COL), fibrin, and agarose (AG) have attracted the most attention as promising matrices for bioinks because of their innate biocompatibility, low cytotoxicity, and high water content, mimicking natural ECM [52,53,54]. SA, which offers fast gelling in the presence of Ca^2+^ or other divalent cations, was frequently used as a bioink for cells to be easily and quickly encapsulated, and for interlayer adhesion during the layer-by-layer printing process [55,56,57]. Studies have indicated that alginate and agarose bioinks support the development of hyaline-like cartilage tissues, whereas GelMA- and PEGMA-based bioinks favor the development of fibrocartilaginous tissues [58,59]. The main disadvantage of chitosan is that it provides poor mechanical integrity to the tissue, making the 3D bioprinted structures brittle and delicate. Finally, the major pitfall with fibrin use is the fast and irreversible gelation at body temperature, which makes its bioprinting difficult [60].

An optical inverted microscope was used for the observation of the differences in cell morphology between the four different cell lines after conducting the MTT colorimetric assay [43]. Similar observations were reported for studies on epithelial cancer cells in 3D culture [61,62,63]. Our results indicated that the immobilization matrix did not affect cell viability. Furthermore, the photometric MTT determination revealed an increase in cellular proliferation relative to the cell population density. On the other hand, when the anticancer agent 5-fluorouracil (5-FU) was added to the cell medium, viability was significantly reduced, suggesting that the 3D immobilization matrix does not influence the influx of the compound in the alginate hydrogel.

After completing the biochemical cytotoxic assays, a bioelectrical analysis by means of impedance measurements was performed on each cancer cell line coupled with its corresponding electrodes in the 3D alginate matrix. The measurement protocol was divided into two cases, based on different aspects of the cell cultures we wanted to investigate. In the first stage, impedance measurements were recorded for different population densities (50,000, 100,000, and 200,000 cells/100 ul) of the aforementioned cell lines in 3D cultures in plain medium. In the second step, the respective measurements were evaluated for 3D cultures after 24 h application of the anticancer compound, 5-FU.

We used the resulting characteristic features to determine contrasts between distinctive cell types by means of normalized impedance magnitudes. The method has been particularly effective in discriminating not only cells of different tissue origin, but also the cytotoxic impact of the anticancer compound.

The method can provide useful information as the assay provides information about the response of cells in specific frequency values, giving us the opportunity to utilize it as a putative cancer diagnostic technique. For further research, this methodology could efficiently be expanded to additional cell lines, i.e., cancerous or normal ones. Similar studies have been conducted on skin [64], breast [65], esophagus [66], and cervical cancer cells [6].

The discrimination method in this study describes a measurement procedure and data processing which is quite easy to handle, although its application for cancer diagnoses or assessments of efficient chemotherapy treatment would require samples that can, in reality, be obtained only by invasive means. Nevertheless, in most cases a definite diagnosis of a malignant tumor depends upon assaying a real tissue sample. That said, the results of the present study demonstrate that even a small number of cells (obtainable through, e.g., liquid biopsy) could be bioelectrically profiled in order to determine their behavior with respect to their susceptibility to selected anticancer agents. While this has not been investigated in the framework of this study, it may be possible to achieve an even higher sensitivity in terms of detecting a low number of cells (i.e., much fewer than 50,000/mL, in the order of a few hundreds or thousands), especially if a considerably wider range of impedance frequencies is used (see also below).

Specific frequency values in the range of 1 KHz–100 KHz were selected to study the electrical properties of the different cell types. Particularly high impedance normalized values were observed at 1 KHz for both treatments, with or without 5-FU, in almost all cell types. At this frequency value, the contribution of cell structure becomes relevant, as the cell-hydrogel interface is influenced by the properties of either the plain cell culture medium and/or the anticancer drug. Hence, cell structure might play an important role in detection sensitivity. For cancerous tissues, a decrease of impedance at frequencies higher than 1 kHz was observed, while in the frequency level up to approximately 100 KHz, these changes were not visible [67]. In addition, it has been reported that the impedance of abnormal tissues, such as breast cancer tissue [68], has lower values compared to those for healthy tissues [69].

Moreover, supplementary investigations could evaluate the prospect of performing a complementary assay on in vivo samples. Our consideration focuses on the fact that these results can begin to highlight the diversities in the electrical behavior of normal and cancerous 3D cell cultures during the whole measurement procedure. The assessment of more heterogeneous models with additional characteristic features, such as resistance or capacitance, could help the enhancement of the system’s resolution capacity. Although our methodology has succeeded in effectively determining various cancer cell types, our next goal is to adjust it to specify the existence of cancer cells in cocultures with normal ones in a single well containing a set of suitably-placed electrodes.

## 5. Conclusions

In this study, we present the proof-of-concept development of a complementary, non-invasive cell analysis method to assess the responses of 3D cultured cancer cell lines derived from various tissues following treatment with cytotoxic concentrations of the well-studied anticancer compound, 5-FU, using bioelectricalal evaluation. A key-feature of the cell-based bioelectrical sensor was the 3D printing of a PETG well, assembled with two gold-coated electrodes, perpendicularly wall-mounted at the bottom, allowing for impedance measurements. The evaluation of our cell bio-system configuration showed good efficacy towards different cell type determination with adequate sensitivity. In order to obtain higher levels of selectivity, and sensitivity based on very low cell populations (up to single-cell analysis), it is necessary to fabricate a different electrode configuration (e.g., screen-printed) which is suitable for single-cell impedance spectroscopy [70,71,72]. As a next step, we plan to conduct more detailed impedance spectral analyses by using a considerably wider continuous frequency range, rather than the discrete values that were used in the present approach, as well as to investigate the effect of different cell immobilization agents on the bioelectric profiling process.

## Figures and Tables

**Table 1:** 5-FU concentrations added to each cell culture.

	SK-N-SH	HEK293	HeLa	MCF-7
5-FU concentration (μΜ)	7.5	20	150	150

**Table 2:** Significant differences (Student’s T-test) between cell populations before and after treatment with 5-FU. **< 0.01, *** < 0.001.

	50,000 Cells	100,000 Cells	200,000 Cells
SK-N-SH	-	**	-
HEK293	-	**	-
HeLa	-	***	-
MCF-7	-	-	-

**Table 3:** Significant differences (Student’s T-test) in cell viability between different cell lines X cell population densities before and after treatment with 5-FU. * < 0.05, **< 0.01, *** < 0.001.

	Cells			Cells Treated with 5-FU		
	50,000 Cells	100,000 Cells	200,000 Cells	50,000 Cells	100,000 Cells	200,000 Cells
SK-N-SH–HEK293	-	-	*	-	-	*
SK-N-SH–HeLa	-	-	**	-	-	-
SK-N-SH–MCF-7	-	*	***	-	***	*
HEK293–HeLa	-	*	-	-	**	*
HEK293–MCF-7	-	*	-	-	**	-
HeLa–MCF-7	-	**	*	-	**	*

**Table 4:** Significant differences (Student’s T-test) between population densities for the SK-N-SH cell line before and after treatment with 5-FU. **< 0.01, *** < 0.001.

	Cells			Cells Treated with 5-FU		
	1 KHz	10 KHz	100 KHz	1 KHz	10 KHz	100 KHz
50,000–100,000	***	***	**	**	***	***
50,000–200,000	-	***	-	***	***	***
100,000–200,000	***	***	-	***	**	***

**Table 5:** Significant differences (Student’s T-test) between population densities for HEK293 cell line before and after treatment with 5-FU. * < 0.05, **< 0.01, *** < 0.001.

	Cells			Cells Treated with 5-FU		
	1 KHz	10 KHz	100 KHz	1 KHz	10 KHz	100 KHz
50,000–100,000	***	***	**	***	***	**
50,000–200,000	***	***	*	***	-	**
100,000–200,000	***	***	***	***	*	**

**Table 6:** Significant differences (Student’s T-test) between population densities for HeLa cell line before and after treatment with 5-FU. * < 0.05, **< 0.01, *** < 0.001.

	Cells			Cells Treated with 5-FU		
	1 KHz	10 KHz	100 KHz	1 KHz	10 KHz	100 KHz
50,000–100,000	***	***	*	***	***	**
50,000–200,000	***	**	***	-	***	***
100,000–200,000	*	***	***	***	***	***

**Table 7:** Significant differences (Student’s T-test) between population densities for MCF-7 cell line before and after treatment with 5-FU. * < 0.05, **< 0.01, *** < 0.001.

	Cells			Cells Treated with 5-FU		
	1 KHz	10 KHz	100 KHz	1 KHz	10 KHz	100 KHz
50,000–100,000	-	***	**	***	***	***
50,000–200,000	**	***	***	***	***	***
100,000–200,000	*	***	*	***	***	**
